# Forensic Evaluation of the Ion AmpliSeq MH-74 Microhaplotype Panel in the Portuguese Population

**DOI:** 10.3390/genes17060628

**Published:** 2026-05-30

**Authors:** Rui Nascimento, Heloísa Afonso Costa, Eugénia Cunha, António Amorim

**Affiliations:** 1 Instituto Nacional de Medicina Legal e Ciências Forenses, 3000-548 Coimbra, Portugal; rui.f.nascimento@inmlcf.mj.pt (R.N.); heloisa.a.costa@inmlcf.mj.pt (H.A.C.); eugenia.m.cunha@inmlcf.mj.pt (E.C.); 2 Escola de Ciências da Vida e Ambiente, Universidade de Trás-os-Montes e Alto Douro, 5000-801 Vila Real, Portugal; 3 Faculdade de Ciências e Tecnologia, Universidade Nova de Lisboa, 2829-516 Caparica, Portugal; 4 Faculdade de Ciências e Tecnologia, Universidade de Coimbra, 3030-790 Coimbra, Portugal; 5 Faculdade de Ciências, Universidade de Lisboa, 1749-016 Lisboa, Portugal; 6 Faculdade de Medicina, Universidade de Lisboa, 1649-028 Lisboa, Portugal; 7 Faculdade de Direito, Universidade Lusófona, 1749-024 Lisboa, Portugal; 8 LAQV REQUIMTE-Associated Laboratory for Green Chemistry and Technology, University of Porto, 4200-465 Porto, Portugal

**Keywords:** microhaplotypes, forensic genetics, massively parallel sequencing, population genetics, kinship analysis

## Abstract

**Background/Objectives**: Microhaplotypes have emerged as promising genetic markers for forensic applications, combining low mutation rates with high polymorphic information. However, population data remain limited for several regions, including Portugal. This study aimed to generate population data for the Ion AmpliSeq™ MH-74 panel in a Portuguese population and evaluate its performance for forensic identification and kinship analysis. **Methods**: A total of 237 unrelated individuals were genotyped using massively parallel sequencing on the Ion GeneStudio S5 platform. Allele frequencies and forensic parameters were estimated. Population structure was assessed using principal component analysis (PCA) based on reference data from MicroHapDB. Kinship performance was evaluated through simulations of full siblings, half siblings, and first cousins and compared with STR markers using likelihood ratios (LR) and error rates. **Results**: The MH-74 panel showed high genetic diversity (mean He = 0.615; mean PIC = 0.565) and strong forensic efficiency, with very high combined power of discrimination and exclusion and a random match probability of 3.86 × 10^−55^. PCA revealed clustering consistent with global population structure, with the Portuguese population grouping within European populations. Microhaplotypes produced higher log_10_(LR) values for related pairs compared to STRs, particularly for closer relationships, with lower false negative rates. **Conclusions**: The MH-74 panel demonstrates high discriminatory power and reliable performance for kinship analysis in the Portuguese population, supporting the use of microhaplotypes as an effective complement to STR markers in forensic genetics.

## 1. Introduction

Forensic human identification has traditionally relied on short tandem repeats (STRs) typed by capillary electrophoresis, which provide high levels of polymorphism and strong discriminatory power. Nevertheless, STR markers present several limitations, including stutter artifacts, relatively high mutation rates compared to other marker systems, and reduced performance when analyzing highly degraded DNA samples [1,2,3].

Microhaplotypes have recently emerged as promising genetic markers for forensic applications. These markers consist of clusters of closely linked single nucleotide polymorphisms (SNPs) located within short genomic regions, typically smaller than 200–300 bp, which form multi-allelic haplotypes [4,5]. Microhaplotypes combine several advantages of SNPs and STRs: they can be amplified from short DNA fragments, do not generate stutter artifacts, and their haplotypic structure increases the effective number of alleles and overall polymorphic information content compared with individual SNP markers [4,5,6].

The development of massively parallel sequencing (MPS) technologies has further increased the forensic utility of microhaplotypes. MPS enables direct phasing of closely linked SNPs and accurate detection of haplotypes, improving their application in mixture deconvolution, ancestry inference, and kinship analysis [6,7,8,9,10,11]. Recent studies have further demonstrated the value of microhaplotypes in complex kinship testing and mixture interpretation, supporting their increasing relevance in forensic casework [12,13]. In particular, the absence of stutter artifacts and the ability to resolve phased haplotypes enhance the interpretation of complex and unbalanced DNA mixtures, representing a significant advantage over traditional STR-based approaches [14,15,16,17]. In parallel, open resources such as MicroHapDB provide curated information on microhaplotype loci and allele frequencies across worldwide populations, facilitating marker selection and comparative population analyses [18].

Several microhaplotype panels have been proposed for forensic purposes using MPS technologies. Among them, the Ion AmpliSeq™ MH-74 Plex Panel (Thermo Fisher Scientific) targets 74 microhaplotype loci, designed for short amplicon sequencing and high allelic diversity, making it particularly suitable for forensic casework [19]. However, reliable population data remain limited for many regions. To date, no population reference data for this panel have been reported for the Portuguese population. Such data are essential for estimating haplotype frequencies and calculating match probabilities and likelihood ratios in forensic genetic analyses.

In this study, we analyzed 237 unrelated individuals from the Portuguese population using the MH-74 panel on the Ion Torrent GeneStudio S5 platform. Haplotype frequencies were estimated and standard forensic parameters were calculated. The Portuguese dataset was further compared with worldwide populations using principal component analysis. In addition, kinship simulations were performed for full siblings, half-siblings, and cousins to evaluate the performance of microhaplotypes in relationship testing beyond direct parent–child comparisons, where conventional STR markers already provide strong evidential support. This study provides the first population dataset for these microhaplotypes in Portugal and supports their applicability in forensic identification and kinship analysis.

## 2. Materials and Methods

### 2.1. Sample Collection

A total of 237 bloodstain samples were analyzed from unrelated individuals residing in Portugal. The samples were collected during routine paternity testing conducted at the National Institute of Legal Medicine and Forensic Sciences (INMLCF), Portugal.

This study was approved by the Ethics Committee of the INMLCF and by the Department of Information, Training, and Documentation of the Institute (Approval number: CE-05/2023). The use of these samples complies with Portuguese legislation (Decree-Law No. 45/2004 of 19 August; Decree-Law No. 12/2005 of 26 January; Decree-Law No. 849/2010), as well as with institutional regulations, which permit the use of biological samples stored for more than two years after casework completion, provided they are fully anonymized.

### 2.2. DNA Extraction and Quantification

Genomic DNA was extracted from bloodstains using the Chelex^®^ 100 protocol as described by Walsh et al. [20]. Samples were concentrated and purified with the Microcon Ultracel YM-100 filters (Milipore Corporation, Burlington, MA, USA). DNA quantification was performed using the Quantifiler™ Trio DNA Quantification Kit (Thermo Fisher Scientific, Waltham, MA, USA), following the manufacturer’s instructions, on a 7500 Real-Time PCR System (Applied Biosystems, Waltham, MA, USA).

### 2.3. Library Preparation

All library preparation steps were performed using an Applied Biosystems^®^ GeneAmp^®^ 9700 96-well thermal cycler (Thermo Fisher Scientific).

Libraries were prepared using the Ion AmpliSeq™ Library Kit 2.0 (Thermo Fisher Scientific), following the manufacturer’s protocol (Ion AmpliSeq™ Library Kit 2.0 User Guide, Thermo Fisher Scientific, rev. H). Briefly, PCR amplification was performed in a total volume of 20 µL, containing 4 µL of 5× Ion AmpliSeq™ HiFi Mix, 10 µL of primer pool (Ion AmpliSeq™ MH-74 Plex Research Panel—Thermo Fisher Scientific) and 1 ng of genomic DNA diluted in 6 µL of molecular-grade water. Thermal cycling conditions were as follows: initial denaturation at 99 °C for 2 min, followed by 22 cycles of 99 °C for 15 s and 60 °C for 4 min. Partial digestion of primer sequences was performed by adding 2 µL of FuPa reagent, followed by incubation at 50 °C for 10 min, 55 °C for 10 min, and 60 °C for 20 min. Adapter ligation was carried out by adding 2 µL of IonCode™ Barcode Adapters (Thermo Fisher Scientific), 4 µL of Switch Solution, and 2 µL of DNA ligase, followed by incubation at 22 °C for 30 min, 68 °C for 5 min, and 72 °C for 5 min.

Libraries were purified using MagMAX™ PureBind™ Beads at a 1.5× ratio, with 70% freshly prepared ethanol, according to the manufacturer’s instructions (Ion AmpliSeq™ Library Kit 2.0 User Guide, Thermo Fisher Scientific, rev. H).

### 2.4. Templating and Sequencing

Libraries were quantified using the Ion Library TaqMan™ Quantitation Kit (Thermo Fisher Scientific), following the manufacturer’s instructions.

Equimolar pooling was performed to a final concentration of 20 pM, according to the laboratory workflow established for the MH-74 panel and routinely used with Ion GeneStudio™ S5, Ion 530™ chips, and Precision ID chemistry (Thermo Fisher Scientific). Template preparation was carried out on the Ion Chef™ System using the Ion S5™ Precision ID Chef & Sequencing Kit (Thermo Fisher Scientific) and Ion 530™ chips. Sequencing was performed on the Ion GeneStudio™ S5 System.

Data analysis was conducted on the Ion GeneStudio™ S5 Torrent Server v5.12.3 using the HID-Microhaplotype-Research-PluginV1.5. The hg19 human reference genome was used together with the manufacturer-provided MH-74 target and hotspot BED files (mh74_target_v1.1 and mh74_hotspot_v1.1). Default plugin thresholds were applied, including a minimum total read coverage of 20 reads per position, a minimum allele count of 5 reads, a minimum allele frequency of 10% for heterozygous calls, and a minimum allele frequency of 90% for homozygous calls. Genotypes were exported from the plugin output and compiled using a custom R script.

### 2.5. Forensic and Statistical Parameters

Population genetic analyses were performed using Arlequin v3.5.2.2 [21], including allele frequencies, observed (Ho) and expected (He) heterozygosity, linkage disequilibrium (LD), and Hardy–Weinberg equilibrium (HWE) testing.

Results from the Arlequin software were independently validated using STRAF v2.2.2 [22], which was also used to calculate forensic parameters, including polymorphism information content (PIC), match probability (PM), power of discrimination (PD), power of exclusion (PE), and typical paternity index (TPI). Combined power of discrimination (CPD) and combined power of exclusion (CPE) were also calculated across loci. As STRAF does not natively support microhaplotype (or SNPs) genotypes, these were converted into a numerical format prior to analysis using a custom R script [23].

### 2.6. Population Data Analysis

Genetic relationships among populations were assessed using principal component analysis (PCA) using allele frequency data. Reference populations were obtained from MicroHapDB and restricted to loci included in the MH-74 panel. Where multiple datasets were available for the same population, selected sources were retained and analyzed independently.

Allele frequencies were arranged into a population-by-allele matrix, and the Portuguese population was incorporated using frequencies estimated in this study.

PCA was performed in R using the “prcomp” function with centering and without scaling. Populations were grouped into seven geographical regions (“Africa, Sub-Sahara”, “South Central Asia”, “East Asia”, “Southwest Asia, Europe”, “Admixed”, “Americas”, and “Pacific”), while the populations included in the analysis are listed in Supplementary Data S1. Visualizations were generated using the “ggplot2” and “plotly” packages [24,25].

### 2.7. Kinship Analysis

Kinship performance was evaluated through simulations of related and unrelated individuals using allele frequencies from the Portuguese population for the MH-74 panel and for the 23 STR markers included in the PowerPlex Fusion 6C Kit (Promega, Madison, WI, USA). The STR allele frequencies used for comparison correspond to unpublished Portuguese population data generated in our laboratory and are part of a manuscript currently in preparation.

Simulations were performed in Familias v3.4 [26,27], with 20,000 iterations conducted for each relationship scenario. The evaluated relationships included full siblings, half-siblings, and first cousins. Simulations assumed Hardy–Weinberg equilibrium and independence between loci. Likelihood ratios (LRs) were calculated under competing hypotheses of related versus unrelated individuals.

False positive and false negative rates were calculated for each relationship scenario at likelihood ratio (LR) thresholds of 1, 10, 100, 1000, and 10,000. False positives were defined as unrelated pairs incorrectly classified as related (LR ≥ threshold), whereas false negatives corresponded to related pairs incorrectly classified as unrelated (LR < threshold). These metrics were used to evaluate the performance of the marker sets across different decision thresholds.

All calculations and graphical representations were performed in R v4.5.2 using ggplot2.

## 3. Results

### 3.1. Genotyping Overview and Allele Frequencies

A total of 237 samples were successfully genotyped, with complete profiles obtained for all individuals across the 74 microhaplotype loci included in the MH-74 panel.

Allele frequencies were estimated for all loci, with the observed number of alleles per locus ranging from 2 to 15, reflecting variability in marker informativeness.

Hardy–Weinberg equilibrium (HWE) testing identified a limited number of loci with nominal deviations (*p* < 0.05); however, none remained statistically significant after Bonferroni correction.

Linkage disequilibrium (LD) analysis identified 13 locus pairs showing statistically significant associations after Bonferroni correction. These included both intra- and interchromosomal associations. The majority of significant LD pairs involved loci located on different chromosomes, suggesting that these associations are unlikely to reflect physical linkage. Among the intrachromosomal pairs, significant LD was observed for mh03KK-150–mh03KK-020, mh05KK-122–mh05KK-123, mh05KK-122–mh05KK-124, mh05KK-123–mh05KK-124, mh17KK-052–mh17KK-105, and mh21KK-315–mh21KK-316.

It should be noted that the Arlequin output reports *p*-values with limited decimal precision (five decimal places), which may result in extremely small *p*-values being displayed as 0.00000. Consequently, the exact magnitude of these *p*-values could not be fully assessed, and some of the reported significant associations should be interpreted with caution.

Complete allele frequency data, as well as HWE and LD results for all loci, are provided in Supplementary Data S2–S4.

### 3.2. Forensic Parameters

The effective number of alleles (Ae), which represents the number of equally frequent alleles that would produce the observed level of genetic diversity at a locus, ranged from 1.026 (mh16KK-053) to 8.282 (mh13KK-218), with a mean value of 3.29. Expected heterozygosity (He) ranged from 0.025 to 0.879, with an overall mean of 0.615 (Supplementary Data S5).

Polymorphism information content (PIC) values ranged from 0.025 to 0.866, with a mean value of 0.565. The power of discrimination (PD) ranged from 0.050 to 0.972, with a mean value of 0.772, while the power of exclusion (PE) ranged from 0.001 to 0.725, with a mean value of 0.350 (Supplementary Data S5).

At the combined level, the MH-74 panel showed very high combined power of discrimination (CPD) and combined power of exclusion (CPE), indicating strong overall performance for individualization and exclusion-based analyses. The combined match probability, corresponding to the random match probability (RMP), was 3.86 × 10^−55^.

### 3.3. Population Analysis

**Figure 1:**
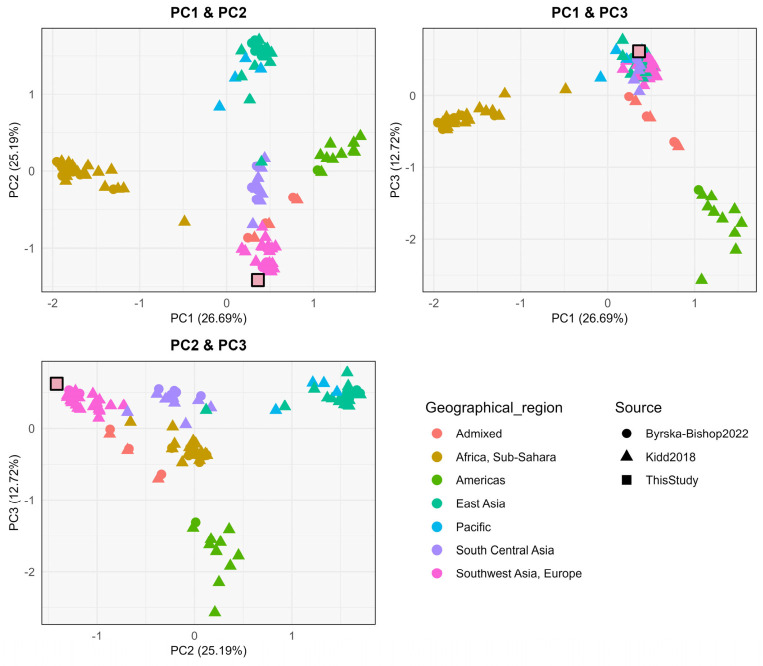
Principal component analysis (PCA) based on microhaplotype allele frequencies for the MH-74 panel. Populations are colored according to geographical region and shaped according to the reference source. The Portuguese population analyzed in this study is highlighted.

Principal component analysis (PCA) based on allele frequency data revealed clustering of populations according to geographical origin (Figure 1).

The first principal component (PC1) explained 26.69% of the total variance, while PC2 and PC3 accounted for 25.19% and 12.72%, respectively.

The Portuguese population clustered within the European group, showing close proximity to other European populations and partial overlap with Southwest Asian populations. Similar clustering patterns were observed across all principal component projections.

A three-dimensional PCA representation was also generated to facilitate visualization of the population structure (Supplementary Data S6).

### 3.4. Kinship Analysis Results

**Figure 2:**
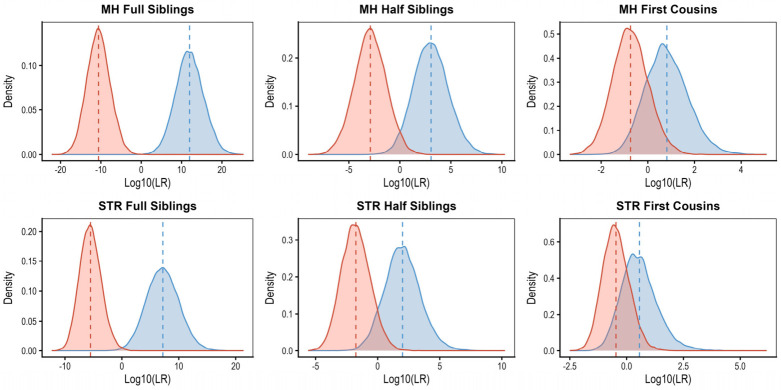
Distribution of log_10_(LR) values for pairwise kinship scenarios involving related and unrelated individuals across different relationship categories (full siblings, half siblings, and first cousins), comparing microhaplotypes (MH-74 panel) and a 23-locus STR panel in the Portuguese population. The *x*-axis represents log_10_(LR) values, and the *y*-axis represents density. Blue curves represent related pairs, and red curves represent unrelated pairs. Vertical lines indicate median log_10_(LR) values for each distribution.

Likelihood ratio (LR) distributions were obtained for full siblings, half siblings, and first cousins (Figure 2 and Supplementary Data S7). For full siblings, LR distributions for related individuals were shifted towards higher values compared to unrelated individuals for both microhaplotypes and STR markers, with minimal overlap between distributions. For half siblings, the separation between related and unrelated distributions decreased, with increased overlap compared to full siblings. For first cousins, substantial overlap between related and unrelated distributions was observed for both marker systems. Microhaplotypes consistently produced higher log_10_(LR) values for related pairs and lower values for unrelated pairs when compared to STRs across all evaluated relationships. Median LR values are summarized in Supplementary Data S8.

False positive and false negative rates varied with the LR threshold. Increasing thresholds reduced false positives but increased false negatives. For full siblings, false positive rates reached 0.00% for both marker systems at LR ≥ 1000, while false negative rates at LR ≥ 10,000 were lower for MH-74 than for STRs (0.99% vs. 12.88%). Higher false negative rates were observed for half siblings and first cousins, particularly at LR ≥ 10,000. Across all scenarios, microhaplotypes showed lower false negative rates compared to STRs (Table 1).

## 4. Discussion

### 4.1. Genetic Diversity and Forensic Efficiency

The microhaplotypes included in the MH-74 panel demonstrated high levels of genetic diversity in the Portuguese population, as reflected by the observed heterozygosity, polymorphism information content, and effective number of alleles. The mean values obtained (He = 0.615; PIC = 0.565) are consistent with those reported in other population studies using the same panel and similar microhaplotype sets, where comparable levels of genetic diversity and marker informativeness have been observed across European and global populations [7,9,28,29,30].

As reported in previous studies, microhaplotype loci show considerable variability in their effective number of alleles (Ae), reflecting differences in haplotype structure and allele frequency distribution [9,11,29,30]. In the present study, Ae values ranged from 1.026 to 8.282, consistent with the wide range described in the literature for panels optimized for forensic applications. Loci with higher Ae values contribute disproportionately to the overall discriminatory power, supporting their relevance in panel design and marker selection. It is also important to consider that the MH-74 panel was not designed solely for individual identification and kinship testing, but rather to support multiple forensic applications, including mixture deconvolution and population-based analyses [13,14,19,29]. The selection of loci aimed to balance high polymorphic information with performance across different populations, taking into account variability in allele frequencies and haplotype structures. Consequently, loci exhibiting lower Ae values in the Portuguese population may display higher levels of diversity in other populations, reflecting differences in population genetic backgrounds. This population-dependent variability has been reported in previous studies using the same panel, where differences in allele frequencies resulted in distinct levels of informativeness across populations. These findings highlight the importance of evaluating microhaplotype panels in diverse populations to fully characterize their forensic utility.

At the combined level, the MH-74 panel exhibited very high forensic efficiency, with very high CPD and CPE values, and a random match probability of 3.86 × 10^−55^. These results are in agreement with previous reports showing that large microhaplotype panels can achieve extremely low random match probabilities, often comparable to or exceeding those obtained with conventional STR systems. This high discriminatory power reflects the multi-allelic nature of microhaplotypes and their ability to capture haplotypic variation within short genomic regions [9,11,29,30].

Overall, the genetic diversity and combined forensic parameters observed in the Portuguese population are consistent with those reported in other populations, supporting the robustness and transferability of the MH-74 panel for forensic identification purposes.

### 4.2. Population Structure and Biogeographical Ancestry

Although the MH-74 panel was not specifically designed for biogeographical ancestry (BGA) inference, the population structure analysis revealed patterns consistent with global genetic variation. Principal component analysis showed clear clustering of major continental groups, with the Portuguese population grouping within the European/Southwest Asian populations.

These results are consistent with previous studies using the same panel and other microhaplotype sets, where European populations typically cluster closely together while maintaining partial overlap with neighboring regions. Similar clustering patterns have also been reported using larger microhaplotype panels, confirming that these markers retain allele frequency differences among populations that are sufficient to reflect broad patterns of population structure despite not being specifically selected for ancestry inference [10,17,29,30].

Microhaplotypes have been shown to provide useful ancestry information when loci are selected based on their informativeness (In), allowing discrimination of multiple continental groups and finer population substructure [10,29]. Although the MH-74 panel was primarily designed for identification and mixture analysis, the observed clustering suggests that it still captures relevant ancestry-related variation.

From a forensic perspective, this additional information may be valuable in investigative contexts, particularly in cases involving unidentified individuals or when generating investigative leads. Furthermore, the ability of microhaplotypes to simultaneously provide information on identity, ancestry, and kinship highlights their versatility compared to single-purpose marker systems.

### 4.3. Kinship Analysis Performance

The evaluation of LR distributions demonstrated that the MH-74 panel provides strong discrimination between related and unrelated individuals, particularly for full sibling relationships. In the Portuguese population, clear separation between related and unrelated distributions was observed for siblings, with high LR values supporting correct classification. Compared with the STR panel, the MH-74 panel produced a clearer separation between related and unrelated LR distributions, particularly for full siblings. This was reflected by higher LR values for true sibling pairs and lower false negative rates across the evaluated thresholds, indicating greater discriminatory power for this relationship category in the Portuguese population. These results are consistent with previous studies using the same panel, where microhaplotypes showed strong performance in distinguishing siblings from unrelated individuals, often with low false-positive rates [9,11].

As shown in Figure 2, the discriminatory power decreased for more distant relationships, such as half siblings and first cousins, where increasing overlap between LR distributions of related and unrelated pairs was observed. Similar patterns have been reported in other studies, where microhaplotype panels provide substantial improvements for close relatives but show reduced resolution for more distant kinship scenarios. Nevertheless, even in these cases, microhaplotypes contribute additional information that may be relevant when combined with other marker systems [9,11].

Importantly, microhaplotypes offer several advantages over conventional STR markers in kinship analysis. Their low mutation rates reduce the occurrence of inconsistencies within families. In addition, the absence of stutter artifacts and the use of short amplicons may improve their performance in degraded samples and complex mixtures. These features are particularly relevant in forensic scenarios such as missing persons identification, where direct parent–child references are often unavailable and indirect kinship inference is required [14,17,31].

In such contexts, the ability to distinguish between siblings becomes especially important, as STR-based approaches may be limited when only collateral relatives are available. The results obtained in the Portuguese population support the usefulness of microhaplotypes in these scenarios, reinforcing their role as valuable markers for biological relationship inference beyond straightforward parent–child testing.

Furthermore, the integration of microhaplotypes with conventional STR markers may enhance the robustness of kinship analyses. While STRs remain highly effective for close relationships, the additional allelic diversity and sequence-based resolution provided by microhaplotypes can improve the overall evidential strength, particularly in complex or deficient kinship cases. This combined approach has been suggested as a practical strategy for implementing massively parallel sequencing data into routine kinship workflows [12,13].

## 5. Conclusions

This study provides the first population dataset for the MH-74 microhaplotype panel in the Portuguese population and demonstrates its high forensic utility. The panel showed high genetic diversity and strong discriminatory power, with combined forensic parameters indicating excellent performance and an extremely low random match probability, supporting its reliability for human identification.

Population analysis confirmed that the Portuguese dataset is consistent with previously described European genetic patterns. Although not specifically designed for ancestry inference, the MH-74 panel retained sufficient allele frequency variation to reflect broad population structure, which may provide additional value in forensic investigations.

In kinship analysis, microhaplotypes showed robust performance, particularly in distinguishing siblings, where clear separation between related and unrelated individuals was observed. Although performance decreased for more distant relationships, the results highlight the added value of microhaplotypes in scenarios where traditional STR-based approaches may be limited.

Overall, these findings support the use of microhaplotypes in the Portuguese population as a complementary tool to STR markers. The integration of both marker systems may enhance the robustness and resolution of forensic investigations, particularly in complex identification and kinship scenarios.

## Figures and Tables

**Table 1:** False positive and false negative rates obtained from simulated data in the Portuguese population for the microhaplotype and STR panels.

Relationship	LR Threshold	False Positives		False Negatives	
		MH-74	STR	MH-74	STR
Siblings	1	0.02%	0.34%	0.01%	0.35%
	10	0.01%	0.04%	0.06%	1.11%
	100	0.00%	0.01%	0.16%	2.99%
	1000	0.00%	0.00%	0.40%	6.63%
	10,000	0.00%	0.00%	0.99%	12.88%
Half-siblings	1	3.21%	6.75%	3.28%	7.80%
	10	0.66%	1.05%	10.90%	24.12%
	100	0.10%	0.13%	27.20%	51.05%
	1000	0.00%	0.01%	49.66%	76.66%
	10,000	0.00%	0.00%	71.55%	91.55%
Cousins	1	16.64%	20.83%	18.73%	24.20%
	10	1.47%	0.99%	59.95%	73.76%
	100	0.05%	0.01%	90.05%	95.41%
	1000	0.00%	0.00%	98.61%	99.36%
	10,000	0.00%	0.00%	99.87%	99.91%

## Data Availability

Data are contained within the article or Supplementary Materials.

## Supplementary Materials

The following supporting information can be downloaded at: https://www.mdpi.com/article/10.3390/genes17060628/s1. Supplementary Data S1: Reference populations included in the population structure analysis. For each population, the abbreviation, population description, and assigned geographical region are provided; Supplementary Data S2: Allele frequency distribution for all 74 microhaplotype loci included in the MH-74 panel in the Portuguese population; Supplementary Data S3: Hardy–Weinberg equilibrium (HWE) results for all loci, including *p*-values and Bonferroni-corrected significance levels; Supplementary Data S4: Pairwise linkage disequilibrium (LD) results for all locus combinations, including *p*-values and Bonferroni-corrected significance levels; Supplementary Data S5: Forensic statistical parameters estimated for each locus, including effective number of alleles (Ae), expected heterozygosity (He), polymorphism information content (PIC), power of discrimination (PD), power of exclusion (PE), and match probability (PM); Supplementary Data S6: Three-dimensional principal component analysis (PCA) based on microhaplotype allele frequencies, including the Portuguese population and reference populations from MicroHapDB; Supplementary Data S7: Distribution of log_10_(LR) values obtained from kinship simulations for full siblings, half siblings, and first cousins, comparing microhaplotypes (MH-74 panel) and a 23-locus STR panel. Solid lines represent related pairs, dashed lines represent unrelated pairs, and vertical lines indicate median values for each distribution; Supplementary Data S8: Median LR values for related and unrelated pairs across different kinship scenarios, comparing microhaplotypes and STR markers. Values are presented in scientific notation when appropriate to facilitate comparison of magnitude.

## Abbreviations

The following abbreviations are used in this manuscript:

Ae	Effective number of alleles
BGA	Biogeographical ancestry
CPD	Combined power of discrimination
CPE	Combined power of exclusion
DNA	Deoxyribonucleic acid
HWE	Hardy–Weinberg equilibrium
LD	Linkage disequilibrium
LR	Likelihood ratio
MPS	Massively parallel sequencing
MH	Microhaplotypes
MH-74	Ion AmpliSeq™ MH-74 microhaplotype panel
PC	Principal component
PCA	Principal component analysis
PCR	Polymerase chain reaction
PD	Power of discrimination
PE	Power of exclusion
PIC	Polymorphism information content
PM	Match probability
RMP	Random match probability
SNP	Single nucleotide polymorphism
STR	Short tandem repeat
