# Supplementary figures and images for: Forensic Evaluation of the Ion AmpliSeq MH-74 Microhaplotype Panel in the Portuguese Population
Source: Genes (Basel). 2026 May 30;17(6):628. doi: 10.3390/genes17060628 (PMC13300383; doi:10.3390/genes17060628)

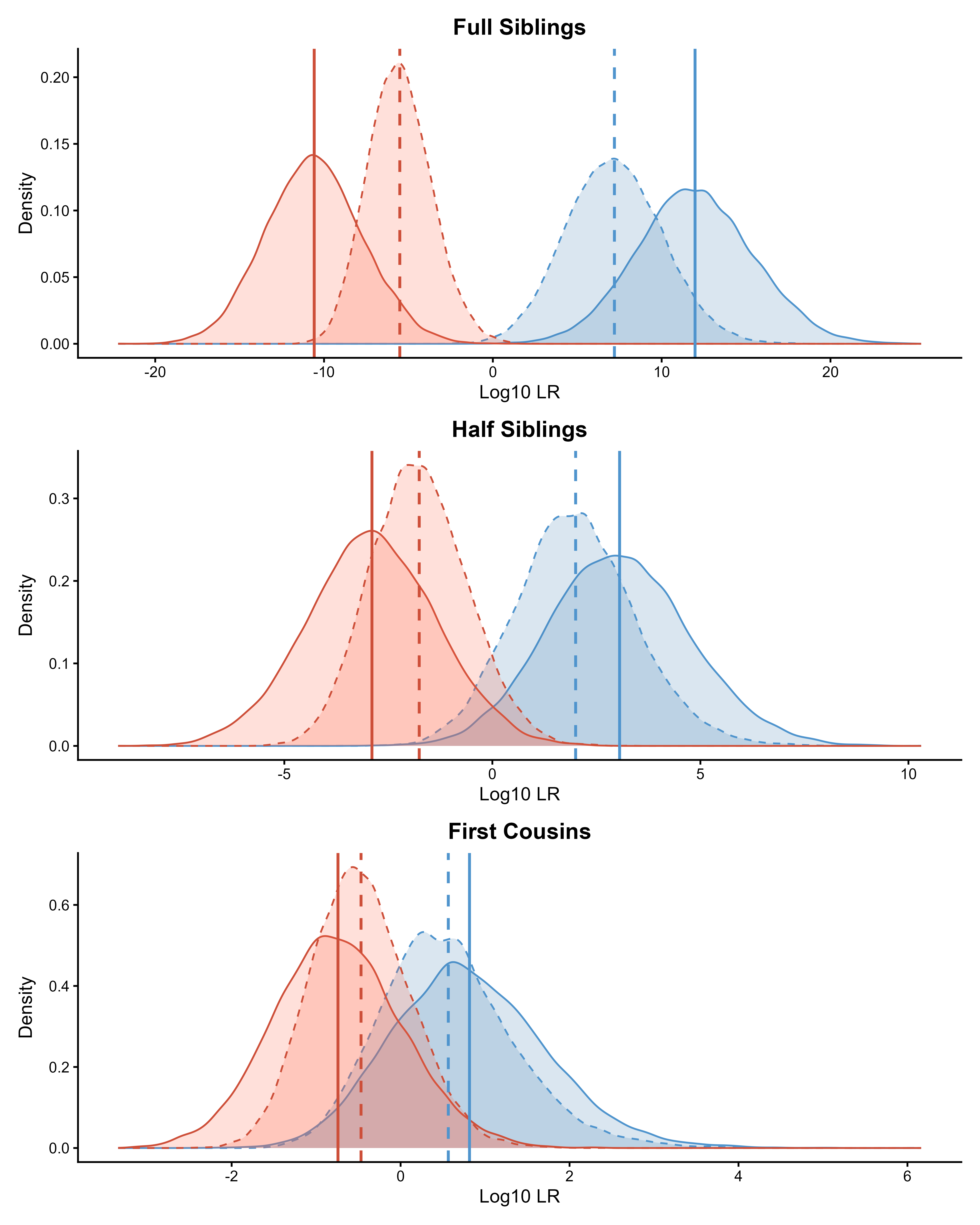

Supplement: Supplementary file 1 [file genes-17-00628-s001.zip › Supplementary_Data_S7_simulations_overlay.png]
